# Parental Warmth, Gratitude, and Prosocial Behavior among Chinese Adolescents: The Moderating Effect of School Climate

**DOI:** 10.3390/ijerph18137033

**Published:** 2021-06-30

**Authors:** Haocheng Luo, Qingqi Liu, Chengfu Yu, Yangang Nie

**Affiliations:** Research Center of Adolescent Psychology and Behavior, Department of Psychology, School of Education, Guangzhou University, Guangzhou 510006, China; luo_haocheng@yeah.net (H.L.); liuqingqi@yeah.net (Q.L.); yuchengfu@gzhu.edu.cn (C.Y.)

**Keywords:** parental warmth, gratitude, prosocial behavior, school climate, adolescent

## Abstract

Parental warmth plays an important role in the development of adolescents’ physical and mental health. There are numerous empirical studies indicating a relationship between parental warmth and prosocial behavior among adolescents, although the underlying mechanisms of this association remain unclear. Adopting a longitudinal design across two time points, the present study proposes a moderated mediation model to explore the mediating role of gratitude and the moderating role of the school climate between parental warmth and prosocial behavior. The sample consisted of 934 participants (483 boys and 451 girls) who participated in the second assessment and completed questionnaires assessing gratitude, school climate, and prosocial behavior in April 2019. Their parents participated in the first assessment and completed a questionnaire pertaining to parental warmth in October 2018. After controlling for the gender and age of the adolescents, the results showed that the positive association between parental warmth and prosocial behavior is mediated by gratitude, and school climate does play a moderating role in the second half of the mediating path. Specifically, the school climate can play a protective role in adolescents with low levels of gratitude. For adolescents with less gratitude, a strong school climate can promote more prosocial behaviors and can effectively alleviate the negative prediction of low levels of gratitude. This study provides a theoretical explanation for the generation of adolescents’ prosocial behavior and provides theoretical guidance for the interventions of schools and parents.

## 1. Introduction

Prosocial behavior refers to all behaviors that individuals make voluntarily to benefit others, including positive and socially responsible behaviors and trends such as helping others, sharing, donation, and self-sacrifice [1]. According to the results of previous research, prosocial behavior can play an important role in adolescents’ lives; adolescents who show more prosocial behavior have better academic performance, physical and mental health, interpersonal relationships, and well-being [2,3,4]. It also has a comprehensive positive impact on the psychological function and social adaptation function of adolescents. Research has shown that, compared to poor prosocial groups, members of good prosocial groups are predicted to display a lower level of externalization behavior [5]. Therefore, considering the positive role of prosocial behavior in adolescents’ individual development, it is necessary to explore the influencing factors and psychological mechanisms of adolescents’ prosocial behavior.

### 1.1. Parental Warmth and Adolescents’ Prosocial Behavior

Parental warmth is an important dimension of parenting style [6] and refers to the degree to which parents intentionally cultivate their children’s personality, self-regulation, and self-assertion by coordinating, supporting, and complying with their children’s needs and requirements [7]. Furthermore, it is a positive family factor and is conducive to the development of adolescents’ empathy abilities and the improvement of their academic achievement [8,9]. Parental warmth is of great significance to the formation of positive psychological traits [10], and it can effectively avoid the development of behavioral problems among adolescents [11]. Bronfenbrenner’s ecosystem theory emphasizes the viewpoint of ecology or system theory, the core of which is to regard development as a process of “individual–environment” interactions [12,13]. For adolescents, family is the environment to which they are directly connected, which will have unequivocal impacts on their behavior. Previous research has shown that parental warmth is conducive to the generation of prosocial behavior in adolescents toward multiple targets, including strangers, family, and friends in school [14]. Adolescents who perceive more warmth from their parents are more likely to develop good psychological traits [10]; adolescents with good psychological traits, such as empathy, will show more positive prosocial behavior in the future [15], which will help them have better academic performance and well-being in future school life [2,3]. Therefore, there is a need to further explore exactly what this trait (or mediating mechanism) is. Additionally, the mediating mechanism could be moderated by other variables. This study was designed to supplement the research gaps in this area.

### 1.2. Gratitude as a Mediator

Gratitude, as one of the positive qualities of adolescents, may mediate the relationship between parental warmth and adolescents’ prosocial behavior. Gratitude refers to a generalized tendency to recognize and respond with grateful emotion to the role of other people’s benevolence in the positive experiences and outcomes that one obtains [16]. Previous studies have shown that parental warmth can affect an adolescent’s gratitude by the mediating role of entitlement and taking perspectives [17]. According to Chandler et al.’s cognitive load theory [18] and Maslow’s hierarchical theory of needs [19], the cognitive resources of adolescents are limited, and adolescents will use their limited cognitive resources to meet their needs. Adolescents with high parental warmth may perceive more care and attention from parents, which may satisfy their emotional and material needs; therefore, they do not need to use their cognitive resources to meet their needs. In contrast, they may devote more cognitive resources to thinking about others, thereby generating more gratitude for others’ kindness and help, instead of allocating cognitive resources to gaining other people’s attention or meeting their own needs.

Adolescents with a higher level of gratitude tend to display more positive prosocial behaviors [20,21,22]. Prosocial behavior is a kind of moral behavior that is highly likely to be triggered by sincere gratitude [23]. Gratitude, as a positive psychological quality, can result in individuals having a healthier psychological state and social adaptability [24], and individuals demonstrating genuine gratitude tend to strengthen their moral behaviors [25]. Previous research has also shown that gratitude improves prosocial behavior by making individuals feel socially valued [20]. Taken together, this theoretical and empirical evidence led to the development of the following hypothesis:

**Hypothesis** **1** **(H1).** *Gratitude will mediate the relationship between parental warmth and adolescents’ prosocial behavior.*

### 1.3. School Climate as a Moderator

Although previous studies have shown that parental warmth has a significant role in reinforcing good adolescents’ prosocial behavior [14], it might not bring about the same effect for all adolescents. Hence, there must be some potential moderators that buffer or aggravate the effect of parental warmth on adolescents’ prosocial behavior. According to Cummings et al.’s organism–environment interaction model and Mischel et al.’s cognitive affective system theory [26,27], individual behavior (e.g., prosocial behavior) is formed and developed in the interactions between the individual and the environment. When adolescents interact with different intrapersonal attributes (e.g., gratitude) and in various environmental contexts (e.g., school climate and parental warmth), they respond differently to their developmental outcomes (e.g., prosocial behavior). The National School Climate Council (2007) defined the school climate as an important microenvironment that influences the physical and mental development of adolescents. The school climate is based on an adolescent’s school life experience and reflects the school’s norms, goals, values, interpersonal relationships, teaching practices, and organizational structure [28].

In the present study, we mainly focused on the influence of teacher–student and student–student relationships on adolescents, because we consider that compared to other factors, teacher–student and student–student relationships play the key influencing roles for Chinese adolescents. The high school climate has been identified by numerous empirical researchers to be a robust positive factor for engendering good adolescent behavior (e.g., prosocial behavior). Luengo Kanacri et al. [29] found that adolescents with higher positive levels of the school climate show a higher level of prosocial behavior in the following year. However, a worse school climate has also been identified to be a robust risk factor for adolescents’ negative behavior. For example, O’Connor et al. [30] found that adolescents who exhibit more aggressive behaviors report lower levels of teacher support.

In addition, some empirical research results have confirmed that the high school climate amplifies the effects of certain factors on adolescents’ positive and negative behavior. For instance, Hou et al. [31] found that the interaction between the average academic achievement of peer group and the perceived teacher support of peer group can positively predict the change of individual academic achievement after half a year. Moreover, Loukas et al. [32] found that interactions between perceptions of cohesion and exerting control are associated with reduced depressive symptoms in boys. Based on the above theoretical framework and empirical evidence, we propose the following hypothesis:

**Hypothesis** **2** **(H2).** *The school climate will moderate the positive indirect link between parental warmth and prosocial behavior. Specifically, the school climate will moderate the direct link and the second half of the indirect link.*

**Figure 1:**
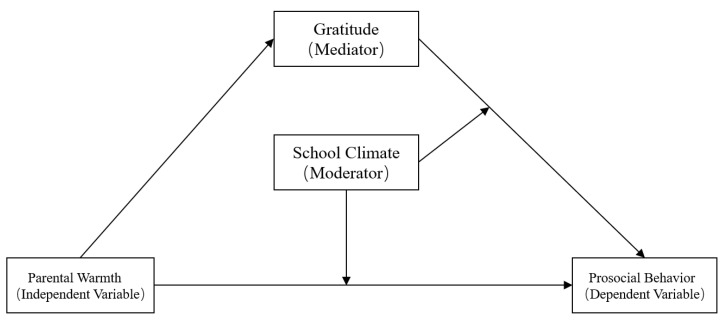
The proposed moderated mediation model.

Grounded in the limited cognitive resource theory and the basic needs theory, this study investigated whether gratitude mediates the relationship between parental warmth and adolescents’ prosocial behavior and whether the second path of this indirect link is moderated by the school climate. Figure 1 illustrates the proposed research model.

## 2. Methods

### 2.1. Participants

The participants in this study included adolescents and their parents who were recruited from five schools, both primary and secondary, in Guangzhou City, Guangdong province, southern China, through stratified and random cluster sampling. A total of 1042 adolescents’ parents participated in the first assessment in October 2018 but just a total of 934 adolescents (51.70% male) ranging in age from 9 to 15 (Mean_age_ = 11.53, *SD* = 3.05) participated in the final assessment in April 2019. The sample mortality was 10.36%. There were 165 students in Grade 4 (17.7%), 195 students in Grade 5 (20.9%), 180 students in Grade 6 (19.3%), 193 students in Grade 7 (20.7%), 105 students in Grade 8 (11.2%), and 96 students in Grade 9 (10.3%), and there was a loss of demographic variables in 330 parents. Only 604 parents’ information were recorded. Among them, 7 families (1.2%) were poor, 47 (7.8%) were not very rich, 381 (63.1%) were in average economic condition, 155 (25.7%) were relatively rich, and 14 (2.3%) were very rich.

### 2.2. Procedure

The research materials and procedures were approved by the ethics committee of Guangzhou University. In this study, a longitudinal study method was used, and data were collected at two time points. Prior to the start of all data collection, we obtained written informed consent from the participants themselves, as well as their parents. The data at time 1 (October 2018) were collected in the students’ homes, and their parents filled in the corresponding questionnaire. The data at time 2 (April 2019) were collected in classrooms by well-trained psychology graduate students and research assistants. Before the formal test, the data collectors informed the participants that participation was voluntary, and if they felt uncomfortable with certain questions, then they did not need to be answered. Participants were assured that their responses would be kept confidential and that they would only be used for academic research.

Mediation and moderation effects were tested with Mplus 8.3 (Muthén and Muthén, Los Angeles, CA, USA) [33]. Missing values were processed by full information maximum likelihood estimation, and bootstrapping analysis with 5000 replicates was performed to verify the significance of the paths. If the confidence interval does not include 0, the path coefficient is significant. According to Hoyle’s suggestion [34], a model fit is considered acceptable when *χ^2^*/*df* is less than 5, CFI and TLI are greater than 0.90, and RMSEA is less than 0.08.

### 2.3. Measures

#### 2.3.1. Parental Warmth

Adolescents’ parents reported parental warmth using the parental warmth questionnaire developed by Teleki et al. [35], with a total of 10 items, such as “I always firmly express my love to my children.” The questionnaire was taken home by adolescents at time 1 and completed by their parents. The parents were asked to report how similar they are to these items on a three-point Likert-type scale, where 1 was “Not like me” and 3 was “Very much like me.” After calculating the average of all items, higher scores meant higher parental warmth. The reliability values obtained in the previous researcher’s application ranged from 0.79–0.89. In this study, the scale demonstrated excellent reliability (α = 0.90).

#### 2.3.2. Gratitude

Adolescents reported their gratitude using the daily gratitude questionnaire compiled by Hussong et al. [36], which was adapted to a certain extent, with a total of 10 items, such as “I used good manners after being given something without being prompted (i.e., say please, thank you).” At time 2, adolescents were asked to report how often they experienced these situations on a six-point Likert-type scale, where 0 was “Never” and 5 was “Always.” After calculating the average of all items, higher scores meant greater gratitude. The reliability values obtained in the original application ranged from 0.79–0.88. In this study, the scale demonstrated excellent reliability (α = 0.85).

#### 2.3.3. Prosocial Behavior

Adolescents reported their prosocial behavior using an adaptation of the Kindness/Generosity Inventory of Strengths [37,38], which was divided into three dimensions, namely, strangers, friends, and family (five items each), with a total of 15 items, such as “Although I don’t think it’s easy, I will help my family.” The questionnaire was completed by the adolescents at time 2 and was scored on a five-point Likert scale, where 1 was “Strongly disagree” and 5 was “Strongly agree.” The higher the average score of all items, the higher the prosocial level of the adolescents. The reliability values obtained in the original application ranged from 0.79–0.93. In this study, the scale demonstrated excellent reliability (α = 0.93).

#### 2.3.4. School Climate

Adolescents reported their perception of the school climate using the school climate questionnaire compiled by Bear et al. [39]. This study used subscales of teacher–student and classmate relationships from the original questionnaire, with a total of nine items, such as “Students get along well.” The questionnaire was completed by the adolescents at time 2, using a four-point Likert scale, where 1 was “Not very true for me” and 4 was “Very true for me.” Higher average scores of all the items meant that the adolescents perceived a better school climate. The reliability values obtained in the original application ranged from 0.91–0.94. In this study, the scale demonstrated excellent reliability (α = 0.92).

#### 2.3.5. Control Variable

We included adolescents’ gender and age as control variables because prior studies have indicated that these variables are significantly associated with prosocial behavior [40]. In this study, the gender and age of the subjects were included in the statistical model for a certain degree of control. The gender of the participants was encoded by dummy variables. All the participants were from five schools in Guangzhou.

### 2.4. Common Method Variance Test

This study controlled the impact of common method bias by two aspects. First, in terms of procedure, we used six months of longitudinal research data, and the data were from parents and adolescents. Second, in order to further enhance the precision of the research, in the statistical analysis, the number of common factors was set as 1, and Mplus 8.3 was used for confirmatory factor analysis (*χ^2^*/*df* = 22.91, CFI = 0.16, TLI = 0.14, RMSEA = 0.15), indicating that there was no serious common method bias in this study [41].

## 3. Results

### 3.1. Preliminary Analyses

In order to describe each variable and the correlation between them, we first used Pearson correlation method for analysis. The means, standard deviations, and correlation coefficients for all of the variables of the current study are displayed in Table 1. The results showed that there was a significant positive correlation between parental warmth, gratitude, and prosocial behavior; school climate was significantly positively correlated with gratitude and prosocial behavior; the school climate had no significant correlation with parents’ emotional warmth. These findings suggest that high levels of parental warmth may be a positive factor of gratitude and prosocial behavior, and a good school climate may be a predictor of prosocial behavior.

### 3.2. Testing for Mediation Effects of Gratitude

**Figure 2:**
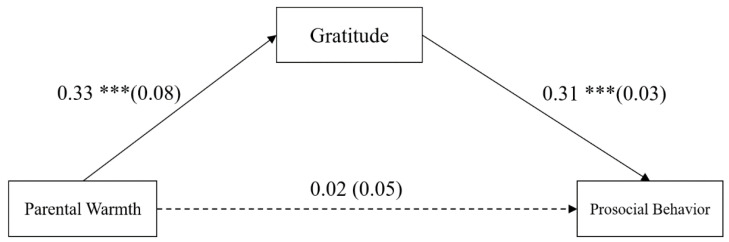
Model of the mediating role of gratitude between parental warmth and prosocial behavior. Values are unstandardized coefficients and the standard error. Paths between gender and age in the model are not displayed because none of these paths were significant. *** *p* < 0.001.

The mediation model represented in Figure 2 revealed an excellent fit to the data: *χ*^2^ = 3.79, *df* = 2, *χ*^2^/*df* = 1.90, CFI = 0.99, TLI = 0.98, RMSEA = 0.03. The results are displayed in Figure 2. Parental warmth positively predicted gratitude (*b* = 0.33, *SE* = 0.08, *p* < 0.001, 95% CI = [0.17, 0.48]) and did not significantly predict prosocial behavior (*b* = 0.02, *SE* = 0.05, *p* > 0.05, 95% CI = [−0.07, 0.12]), although gratitude did positively predict prosocial behavior (*b* = 0.31, *SE* = 0.03, *p* < 0.001, 95% CI = [0.254, 0.372]). Moreover, bootstrapping analyses indicated that gratitude completely mediated the relationship between parental warmth and adolescents’ prosocial behavior (indirect effect = 0.10, *SE* = 0.03, 95% CI = [0.06, 0.16]).

### 3.3. Testing for Moderated Mediation

**Figure 3:**
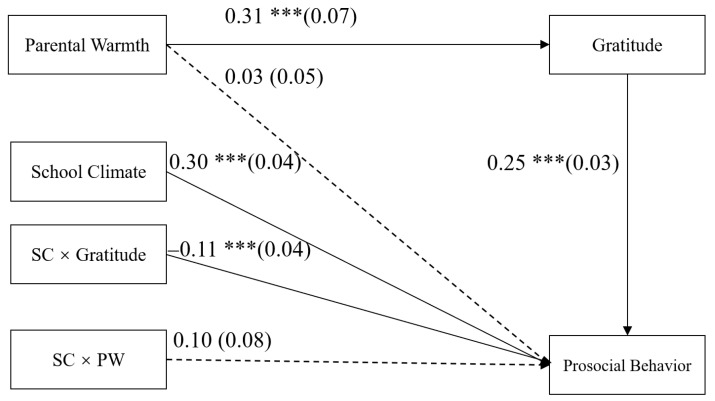
Model of the moderating role of school climate on the indirect relationship between parental warmth and prosocial behavior. SC, school climate; PW, parental warmth. Values are unstandardized coefficients and the standard error. Paths between gender and age in the model are not displayed because none of these paths were significant. *** *p* < 0.001.

The moderated mediation model represented in Figure 3 displayed a good fit to the data: *χ*^2^ = 14.69, *df* = 8, *χ*^2^/*df* = 1.84, CFI = 0.99, TLI = 0.98, RMSEA = 0.03. The bias-corrected percentile bootstrap results indicate that the indirect effect of parental warmth on adolescents’ prosocial behavior through gratitude was moderated by the school climate. Specifically, school climate moderated the association between gratitude and prosocial behavior (*b* = −0.11, *SE* = 0.04, *p* < 0.01, 95% CI = [−0.18, −0.02]). However, school climate did not moderate the association between parental warmth and prosocial behavior (*b* = 0.10, *SE* = 0.08, *p* > 0.05, 95% CI = [−0.06, 0.26]).

**Figure 4:**
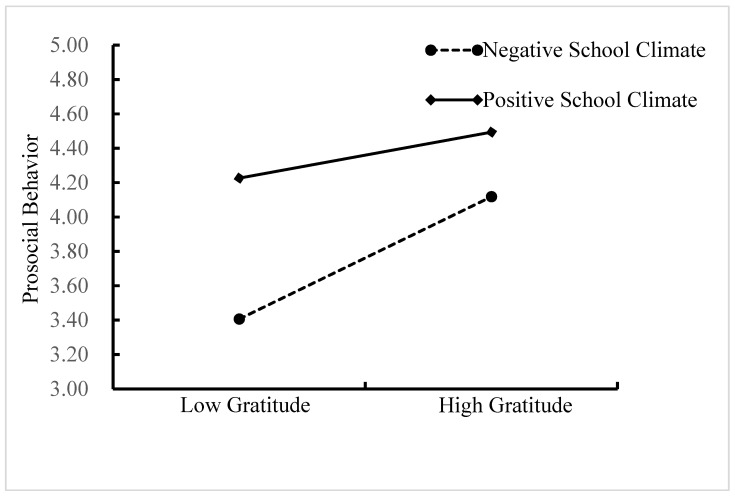
Gratitude among adolescents as a function of prosocial behavior and school climate.

A simple slopes test was conducted in this study, and, as depicted in Figure 4, the positive link between gratitude and prosocial behavior was much stronger for adolescents with a negative school climate (1 *SD* below the mean; *b* = 0.37, *SE* = 0.08, *p* < 0.001, and 95% CI = [0.22, 0.52]) than for adolescents with a positive school climate (1 *SD* above the mean; *b* = 0.24, *SE* = 0.08, *p* < 0.01, and 95% CI = [0.08, 0.39]).

Moreover, the indirect links between parental warmth and prosocial behavior via gratitude were stronger for adolescents with a negative school climate (indirect effect = 0.09, *SE* = 0.02, *p* < 0.001, 95% CI [0.05, 0.14]) than for adolescents with a positive school climate (indirect effect = 0.06, *SE* = 0.02, *p* < 0.01, 95% CI = [0.02, 0.10]).

## 4. Discussion

This study examined how parental warmth relates to adolescent prosocial behavior and whether the association varies according to school climate. This investigation found that adolescents with higher parental warmth show more gratitude, which further relates to improved prosocial behavior. Moreover, this indirect link is moderated by adolescents’ school climate. The present findings enhance our understanding of how parental warmth relates to adolescent prosocial behavior and contributes to identifying underlying mechanisms for prosocial adolescent behavior.

### 4.1. The Mediating Role of Gratitude

The first goal of this study was to explore the mediating effect of gratitude on the effect of parental warmth on adolescents’ prosocial behavior. This study found that gratitude completely mediated the relationship between parental warmth and adolescents’ prosocial behavior, which is consistent with Hypothesis 1. The findings of this study showed that parental warmth helps to increase adolescents’ gratitude, and gratitude further promotes the incidence of adolescents’ prosocial behavior. Based on Chandler et al.’s cognitive load theory [18] and Maslow’s hierarchical theory of needs [19], when adolescents perceive more warmth from their parents’, they are more likely to form good internal psychological traits and devote more cognitive resources to others who help and appreciate them, rather than to their own interests. From the perspective of the Bronfenbrenner’s ecosystem theory [13], family is at the core of the microsystem of adolescent development [42]. Positive parenting styles (parental warmth), such as positive communication, understanding, and tolerance, are conducive to the development and formation of adolescent gratitude, whereas negative parenting styles lead to a lack of emotional communication between parents and children; this is not conducive to the formation of good habits or the development of gratitude [43].

Previous studies have also shown that adolescents with good gratitude are more likely to adopt moral behaviors, such as prosocial behaviors [25], in order to experience stronger feelings of self-efficacy and social worth [20]. It is consistent with the results of this study. Adolescents with high levels of gratitude are more likely to return favors and help others, and they are more likely to perform prosocial actions for others. Combined with the above research and the results of this study, parental warmth does shape at least one excellent quality (gratitude) of adolescents, and adolescents with high levels of gratitude pay more attention to the needs of others and perform more prosocial actions to experience the feelings of self-efficacy and social worth. In summary, gratitude is an important psychological mechanism, where parental warmth affects adolescents’ prosocial behaviors.

### 4.2. The Moderating Role of School Climate

The second goal of this study was to explore moderating effects of the school climate on the indirect association between parental warmth and prosocial behavior via gratitude. The results showed that the school climate significantly moderates the second half of the mediating path, which is partially consistent with Hypothesis 2. This study found that school climate can play a protective role in adolescents with low levels of gratitude. For adolescents displaying less gratitude, a positive school climate can promote more prosocial behaviors and can effectively alleviate negative predictions of low levels of gratitude. Specifically, the positive association between gratitude and prosocial behavior was stronger among adolescents with a negative school climate. However, the protective effect of a positive school climate was more obvious among adolescents with low levels of gratitude. School climate did not moderate the direct link between parental warmth and prosocial behavior. This is because parental warmth mainly works through mediating the role of gratitude instead of a direct effect; thus, direct effects are not moderated by the school climate.

According to Cummings et al.’s organism–environment interaction model and Mischel et al.’s cognitive affective system theory [26,27], individual behavior is induced by interactions of the situation (school climate) and personal factors (gratitude). Adolescents with high levels of gratitude were more inclined to display more prosocial behaviors and were less affected by the school climate; for adolescents with low levels of gratitude, their impulses to perform prosocial actions were weak, and they were more vulnerable to the influence of external environmental factors (i.e., the school climate). This is in line with the peer cluster theory [44]: Adolescents tend to act in accordance with their peers in order to gain acceptance from the group. For adolescents with low levels of gratitude, if they were in a positive school climate, i.e., where they were surrounded by more prosocial peers, their behavior would be more prosocial. In contrast, if they were in a negative school climate, their inclination for prosocial behavior would be reduced. Therefore, the role of the school climate is more obvious for adolescents with less gratitude. The results of this study are also consistent with previous studies. For example, Yang et al. found that the positive association between social awareness and bullying victimization and the negative association between self-management and bullying victimization were both mitigated in schools with more positive school climate [45]. All these show that positive school climate is of great significance to the development of adolescents.

### 4.3. Practical Implications

The results of this study have important theoretical significance and practical value. Our research results show that gratitude is an important mediator of parental warmth affecting adolescents’ prosocial behavior, and school climate plays a moderating role in the second half of this mediating path. Therefore, parents should pay attention to the influence of their parenting style on children. More attention should be paid to emotional warmth and communication with children in an understanding way to cultivate children’s gratitude. In addition, positive school climates do not amplify the positive impacts of parental warmth on prosocial behavior through gratitude, although still have practical significance. The indication is that adolescents with low levels of gratitude are more vulnerable to the influence of the school climate, suggesting that schools and teachers should pay attention to the cultivation of a positive school climate. Teachers and schools can provide adolescents with a better learning and living environment by improving the school climate, which would be more conducive for adolescents to develop more prosocial behaviors. More importantly, this study shows that the interrelationships between schools and families play a key role in the development of adolescents. This is of great significance for future education.

### 4.4. Limitations

This study has some notable limitations, however. First, this study used a self-report method to collect data. Although the method of combining parents’ and children’s reports and longitudinal data was used to control the common method variance to a certain extent, more processing would be needed in future research to further control the common method variance. Second, this study only controlled the gender and age of adolescents. Future research should consider other relevant control variables. Finally, the data in this study only came from two periods of time, which therefore cannot decisively confirm a causal relationship.

## 5. Conclusions

In conclusion, we proposed a moderated mediation model and tested it using a longitudinal method to explore the psychological mechanism between parental warmth and adolescents’ prosocial behavior. Overall, gratitude plays a complete mediating role in the relationship between emotional warmth from parents and prosocial behavior. Parental warmth can promote adolescents’ gratitude, which, in turn, promotes prosocial behavior. Moreover, the school climate plays a moderating role in this mediating relationship. For adolescents with low levels of gratitude, protective effects of the school climate are stronger. These novel findings emphasize the significance of both environmental and individual factors to better understand the incentive of adolescents adopting prosocial behavior.

## Figures and Tables

**Table 1:** Descriptive statistics and correlations for all variables.

Variables	*M*	*SD*	1	2	3	4	5	6
1. Gender	0.52	0.50	1					
2. Age	11.53	3.05	0.36	1				
3. Parental Warmth	2.50	0.44	0.12	−0.09 **	1			
4. Gratitude	3.62	0.95	−0.44	−0.06	0.15 ***	1		
5. Prosocial Behavior	4.12	0.64	0.01	0.01	0.08 *	0.47 ***	1	
6. School Climate	3.26	0.58	0.06	−0.03	0.04	0.33 ***	0.40 ***	1

Note: Gender was dummy-coded: 1 = male, 0 = female; * *p* < 0.05, ** *p* < 0.01, *** *p* < 0.001.

## Data Availability

The data presented in this study are available on request from the corresponding author.
